# Regulatory T Cells Control Vascular Adhesion Molecule Expression in Skin Under Inflammatory and Homeostatic Conditions

**DOI:** 10.1111/micc.70017

**Published:** 2025-06-29

**Authors:** M. Ursula Norman, Brandon Lim, Lucinda Jenkins, Pam Hall, Sarah L. Snelgrove, Michael J. Hickey

**Affiliations:** ^1^ Centre for Inflammatory Diseases, Monash University Department of Medicine Monash Medical Centre Clayton Victoria Australia

**Keywords:** adhesion molecule, dermatitis, endothelium, microvasculature, regulatory T cell

## Abstract

**Objective:**

During skin inflammation, inhibition of adhesion of regulatory T cells (Tregs) to the dermal microvascular endothelium leads to exacerbation of inflammation, evidence that the dermal endothelium is a key target of the anti‐inflammatory actions of Tregs. The aim of this study was to investigate the capacity of Tregs to control the expression of endothelial adhesion molecules in inflamed and resting skin.

**Methods:**

Treg function was assessed in a two‐challenge contact hypersensitivity (CHS) model, measuring dermal adhesion molecule expression via imaging of cleared skin. Treg depletion was achieved using *Foxp3* ^*DTR*^ mice.

**Results:**

CHS induced upregulation of E‐selectin and ICAM‐1 but not P‐selectin and VCAM‐1. Elimination of Tregs following CHS challenge resulted in exacerbated skin inflammation and enhanced expression of E‐selectin, P‐selectin and ICAM‐1 in the dermal microvasculature. Multiphoton imaging revealed that at this phase of the response, Tregs were enriched near blood vessels and underwent dynamic migration adjacent to the microvasculature. Additionally, in skin that was not undergoing hapten challenge, absence of Tregs also resulted in upregulation of E‐selectin and ICAM‐1 in skin vessels.

**Conclusions:**

These observations demonstrate that the microvascular endothelium is a target of the anti‐inflammatory actions of Tregs in the skin, both during CHS and in steady‐state skin.

**Abbreviations**
CHS: contact hypersensitivity
CLA: cutaneous lymphocyte antigen
DT: diphtheria toxin
DTR: diphtheria toxin receptor
iTregs: induced Tregs
mAb: monoclonal antibody
MP‐IVM: multiphoton intravital microscopy
MPM: multiphoton microscopy
Tregs: regulatory T cells

## 1 Introduction

CD4^+^ regulatory T cells (Tregs) play an essential role in suppressing inappropriate inflammation and autoimmune responses in the skin and maintaining skin homeostasis [1, 2]. This is exemplified by the severe skin inflammation seen when these cells are absent or dysfunctional [3, 4, 5]. Healthy skin contains a robust population of Tregs and these cells are continually recirculating through the skin via the vascular and lymphatic systems [6, 7, 8, 9]. However, if Tregs are unable to enter or be retained in the skin, this is sufficient to result in local inflammation [10, 11, 12, 13, 14], while Tregs also control skin inflammation induced by exogenous stimuli [15, 16, 17, 18]. Tregs utilize an array of mechanisms to mediate their anti‐inflammatory effect, many of which target pro‐inflammatory immune cells [19, 20]. However, a growing body of evidence indicates that the vascular endothelium may serve as an additional less well‐recognized target of the actions of Tregs.

Inflammatory conditions of the skin are characterized by recruitment of a wide variety of immune cells that enter the skin via the dermal microvasculature. The critical role of the microvascular endothelium in this process is demonstrated by the attenuation of inflammation in mice lacking endothelial adhesion molecules [21, 22, 23]. Tregs can suppress inflammatory immune cell recruitment in CHS and other conditions raising the possibility that these effects may be mediated via an effect on the endothelium [15, 16, 17, 24, 25, 26, 27]. To that end, in vitro studies of the effects of isolated Tregs on endothelial cells have shown that Tregs can restrict adhesion molecule upregulation by endothelial cells and suppress T cell adhesion and transmigration across endothelial cell monolayers [27, 28, 29]. In addition, recent studies in lung cancer show that endothelial cells promptly modify gene expression in response to removal of Tregs, responding more rapidly than T cells, the best‐characterized targets of Treg suppressive function [30]. These observations support the concept that the microvascular endothelium is an important target of Treg regulatory function.

One form of CHS in which regulation of inflammation is highly active is multiple challenge CHS [31, 32]. In this model, when the same area of skin is re‐challenged after 48 h, when the initial inflammatory response has resolved, inflammation peaks significantly earlier at 4 h versus 24 h after a single challenge, and also resolves more rapidly [31, 32, 33]. This alteration in the response is associated with increased abundance of Tregs in the skin compared to that at the initial challenge. In this two‐challenge form of CHS, blockade of Treg‐endothelial cell interactions results in increased neutrophil adhesion in skin blood vessels [33, 34, 35]. These observations are consistent with Tregs having an inhibitory effect on endothelial adhesion molecule expression in multiple challenge CHS, although this remains to be demonstrated. Therefore, the aim of this study was to investigate the capacity of Tregs to suppress endothelial adhesion molecule expression during multiple‐challenge CHS. Using a novel tissue clearing‐based technique for investigation of adhesion molecule expression in the dermal microvasculature, these experiments revealed that Tregs suppress endothelial upregulation of specific adhesion molecules during CHS and under steady‐state conditions.

## 2 Materials and Methods

### 2.1 Mice

C57BL/6 wildtype mice were obtained from the Monash Animal Research Platform at Monash University. *Foxp3* ^*tm3(DTR/GFP)Ayr*^ (*Foxp3* ^DTR^) mice on a C57BL/6 background were obtained from Jackson Laboratories [36]. *Foxp3* ^DTR^ and B6N.129(Cg)‐Foxp3tm3Ayr/J (Foxp3‐GFP) mice [33] were bred in‐house under specific pathogen‐free conditions. All experiments using animals were approved in advance by the Monash Medical Centre Animal Ethics Committee B. Mice aged between 8 and 12 weeks old were used for all experiments.

### 2.2 Antibodies and Reagents

The following antibodies and reagents were used in this study: anti‐CD31‐AF594 (clone MEC13.3, BioLegend, San Diego, CA); RME‐1, a mAb against rat and mouse E‐selectin, and RMP‐1, a mAb against rat and mouse P‐selectin, were produced in the laboratory of Dr. Andrew Issekutz (Dalhousie University, Halifax, NS, Canada) or purchased (BioLegend, San Diego, CA); YN1.1.7.4, a mAb against mouse ICAM‐1, and 6C7.1, a mAb against murine VCAM‐1, were grown in‐house (6C7.1 hybridoma provided by Dr. Dietmar Vestweber, Max Planck Institut, Meunster, Germany and Dr. Britta Engelhardt, Theodor Kochor Institute, Bern, Switzerland). Isotype controls included mouse IgG1κ (BioLegend) and rat IgG2b (BioXCell, Lebanon, NH). Unconjugated antibodies were conjugated to either Alexa Fluor 488 (AF488) or Alexa Fluor 680 (AF680) using the respective Alexa Fluor antibody labelling kits (Thermo Fisher Scientific, Waltham, MA).

### 2.3 Oxazolone Induced Model of Contact Hypersensitivity

A two‐challenge model of oxazolone‐induced CHS was induced as previously described [33, 34, 35]. Mice were sensitized by application of 50 μL of 5% oxazolone (Sigma‐Aldrich, St Louis, MO) in acetone/olive oil (4:1) vehicle to a shaved area on the back. Five to seven days later, mice were challenged with 50 μL of a 1% oxazolone/acetone/olive oil solution to a shaved area (1 × 2 cm) on the right abdominal flank skin. Mice subsequently underwent a second oxazolone challenge 48 h after the initial challenge, on the same region of skin. In experiments involving assessment of skin swelling, the right ear was challenged (20 μL of the 1% oxazolone solution) at the same time, while the left was treated with vehicle alone. Skin was examined 4 h after the second challenge. Swelling was determined by measuring the thickness of the challenged ear using a micrometer and subtracting the thickness of the vehicle‐treated ear [35].

### 2.4 Treg Depletion Using *Foxp3* ^DTR^ Mice

Treg depletion was achieved using *Foxp3* ^DTR^ mice in which administration of diphtheria toxin (DT) results in rapid and near complete depletion of Tregs [36]. As the aim of these experiments was to focus on the role of Tregs in the challenge phase rather than their actions during sensitisation, DT treatment (50 μg/kg, i.p in PBS) was not initiated until 24 h after the first oxazolone challenge. This protocol ensured that the initial inflammatory response to hapten challenge, which is important for recruitment of the normal leukocyte infiltrate into the inflamed skin, occurred without any effect stemming from absence of Tregs. Mice received a second dose DT at 48 h, at the time of the second challenge. All DT‐treated mice were tested for Treg depletion, by assessing the abundance of Tregs in blood samples via flow cytometry, identifying Tregs on the basis of GFP expression, co‐expressed with the DTR transgene in this strain [36]. Only mice with effective Treg depletion were used in experiments.

### 2.5 Adhesion Molecule Staining and Skin Clearing

Four hours after the second skin challenge, mice were prepared for adhesion molecule staining, using a protocol adapted from Klingberg et al. [37]. In brief, mice were anesthetized via i.p. injection of 150 mg/kg ketamine hydrochloride (Troy Laboratories, Smithfield, NSW, Australia) and 10 mg/kg xylazine (Pfizer, West Ryde, NSW, Australia), and the jugular vein cannulated for antibody administration. Regions of skin intended for collection were treated with depilatory cream. Mice received one of the two following cocktails i.v.: anti‐CD31‐AF594 (10 μg), anti‐E‐selectin‐AF680 (100 μg), anti‐ICAM‐1‐AF488 (50 μg); or anti‐CD31‐AF594 (10 μg), anti‐P‐selectin‐AF680 (100 μg), anti‐VCAM‐1‐AF488 (90 μg). Doses of the selectin and ICAM‐1/VCAM‐1 antibodies were previously determined to saturate available receptors [38, 39]. For control staining experiments, isotype control antibodies for the selectin (mouse IgG1) and ICAM‐1 (rat IgG2b) antibodies were conjugated to the corresponding fluorochromes and administered to mice in the same manner. Antibodies were allowed to circulate for 10 min. The mouse was subsequently euthanized via anesthetic overdose and then immediately exsanguinated via transcardiac perfusion with PBS/5 mM EDTA (15 mL) followed by perfusion fixation with 4% paraformaldehyde in PBS (15 mL). Regions (1 cm^2^) of skin from the challenge site and an additional region of uninvolved abdominal skin were removed and post‐fixed in 4% paraformaldehyde for 2 h. Samples were subsequently dehydrated through graded ethanol solutions (pH 9.0, 4°C) and transferred to ethyl cinnamate at room temperature for a minimum of 2 h prior to imaging. For imaging, samples were mounted on a slide in a well of vacuum grease filled with ethyl cinnamate and covered with a coverslip.

### 2.6 Image Acquisition

Cleared skin samples were imaged via MPM. MPM was performed with an Olympus FVMPE‐RS microscope equipped with a 25 × 1.05 NA lens, four non‐descanned detectors and a multiphoton laser (Insight X3, *Spectra Physics*, NewSpec, Myrtle Bank, SA, Australia). AF488 and AF594‐conjugated antibodies were imaged at 810 nm and AF680‐conjugated antibodies were imaged at 900 nm excitation. Pilot experiments in samples containing AF488‐, AF594‐ and AF680‐conjugated antibodies revealed that illumination at 900 nm excited AF680 but not the potentially overlapping AF594. As such investigation of AF680 staining was performed exclusively using 900 nm excitation, ensuring that the signal captured under these conditions was specific to AF680.

Images were collected at a resolution of 512 × 512 pixels, 3 μm *z*‐step size, 1.5 zoom, to typical depths of up to 700 μm, across a randomly selected region encompassing 3 × 3 adjacent fields. Images of a total area of 1017 × 1017 μm (*x, y*) were acquired. For displaying the data, images are maximum intensity projections through the skin, taken from the epithelial surface to ~400 μm depth in inflamed samples, and ~ 200 μm in non‐inflamed samples.

### 2.7 Image Analysis

Adhesion molecule expression in acquired skin images was analyzed using *Imaris* image analysis software (v 9.2.1, Oxford Instruments, Abingdom, UK). The automated surface tool algorithm, with manual correction to remove surfaces from hair follicles and fluorescence artifacts, was used to apply surfaces to stained regions of the vasculature and subsequently calculate the surface area of adhesion molecule expression in each volume of tissue. First the total vessel surface area was defined based on CD31 staining of the vasculature. Subsequently the area occupied by staining for each of the adhesion molecules within the vasculature was determined and expressed as μm^2^/field of view. Analysis was performed for 9 imaged volumes of skin in each sample and the data averaged across the samples.

### 2.8 Multiphoton Intravital Microscopy of Inflamed Skin

MP‐IVM of flank skin undergoing the CHS response was performed as previously described [40, 41, 42]. In brief, mice were anesthetized via ketamine/xylazine cocktail. Body temperature was maintained using a heat pad. A midline incision was made in the abdominal skin and the flank skin extended over a heated pedestal with the epidermal side facing up. The exposed area was immersed in saline and enclosed with a coverslip held in place by vacuum grease.

MP‐IVM of flank skin of Foxp3‐GFP mice was performed with an Olympus FVMPE‐RS microscope equipped with a 25 × 1.05 NA lens, four non‐descanned detectors and a multiphoton laser (Insight X3, *Spectra Physics*, NewSpec, Myrtle Bank, SA, Australia). Experiments were performed at 900 nm excitation. To enable visualization of the vasculature, AF680‐albumin (25 μg/mouse, Thermo Fisher Scientific) was administered i.v. Images were collected at 512 × 512 resolution and 2 μm *z*‐step size, to depths of 100–150 μm, acquired every 60 s for a total of 30 min per recording. Typical experiments involved 3 recordings of non‐overlapping regions of the flank skin. Behavior of GFP^+^ Tregs was analyzed using *Imaris* via the automated surface tool algorithm (with manual correction), determining track displacement (μm), track length (μm), mean velocity (μm/min), confinement (displacement/track length) and nearest distance to AF680‐albumin‐labeled vasculature.

### 2.9 RNA Isolation, cDNA Synthesis and RT‐qPCR

1 cm^2^ samples were obtained from the shaved flanks of CHS‐challenged C57BL/6 mice, flash frozen in liquid nitrogen and stored at −80°C until use. RNA isolation was performed as described previously [40]. Gene expression by qPCR was performed using a Taqman gene expression assay (gene and assay numbers shown in Table S1). All data were normalized to the housekeeping gene *Actb* and are expressed using the 2^−ΔΔCT^ method to compare expression to the averaged 2^−ΔCT^ isotype control‐treated group.

### 2.10 Statistics

GraphPad Prism software was used for all statistical analysis. Where comparison was made between treated and control samples from the same animal, paired 2‐tailed *t*‐tests were used. Where samples were derived from two separate groups of mice, unpaired 2‐tailed *t* tests were used. Significance was defined as *p* < 0.05.

## 3 Results

### 3.1 Tregs Control Inflammation in Two‐Challenge CHS

**FIGURE 1:**
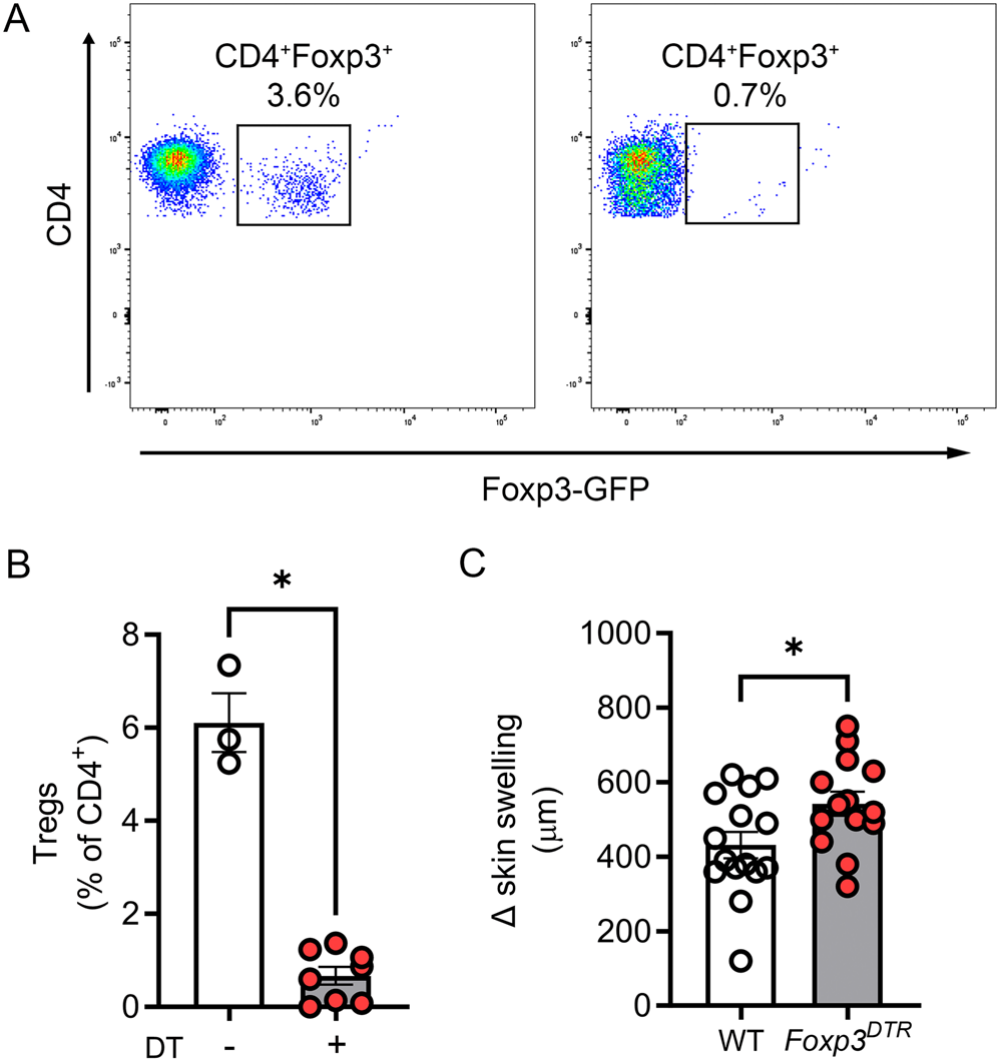
Treg depletion exacerbates skin inflammation in two‐challenge CHS. Oxazolone‐sensitized *Foxp3* ^DTR^ mice were treated with diphtheria toxin (DT) commencing 24 h after a first oxazolone challenge. (A, B) Treg depletion measured 4 h after a second challenge. (A) Representative flow cytometry plots showing blood CD4^+^ T cells in an untreated *Foxp3* ^DTR^ mouse (left panel) and a DT‐treated *Foxp3* ^DTR^ mouse (right panel). Treg abundance is shown as percentage of CD4^+^ T cells, after identification of Tregs via GFP expression in *Foxp3* ^DTR^ mice (representative data from *n* = 8 *Foxp3* ^DTR^ mice). (B) Group data comparing % of Tregs in *Foxp3* ^DTR^ mice in the presence or absence of DT treatment. (C) Skin swelling 4 h after a second oxazolone challenge in DT‐treated wild‐type (WT) and *Foxp3* ^DTR^ mice. Data are shown as mean ± sem, with data for individual mice also shown. In (B), no DT—*n* = 3, DT‐treated—*n* = 8. In (C), WT—*n* = 15, *Foxp3* ^DTR^—*n* = 14. *p < 0.05 for comparisons shown.

Tregs have been shown to suppress inflammation in a single‐challenge CHS model [17], although this has not been investigated in the two‐challenge model. Therefore, we first sought to clarify the contribution of Tregs in CHS induced by two oxazolone challenges, making use of *Foxp3* ^DTR^ mice in which DT administration causes Treg depletion [36]. To ensure the effects of Treg depletion were restricted to the challenge phase [17], administration of DT was not commenced until 24 h after initial hapten challenge. Despite this, almost complete Treg depletion was achieved 4 h after the second challenge (28 h after initiating DT treatment), both in the circulation (Figure 1A,B) and skin (data not shown). Treg depletion in two‐challenge CHS led to significant exacerbation of skin swelling (Figure 1C), demonstrating that the anti‐inflammatory effects of Tregs in this model were comparable to those previously observed in single‐challenge CHS.

### 3.2 Two Challenge CHS Causes Upregulation of E‐Selectin and ICAM in the Microvasculature

**FIGURE 2:**
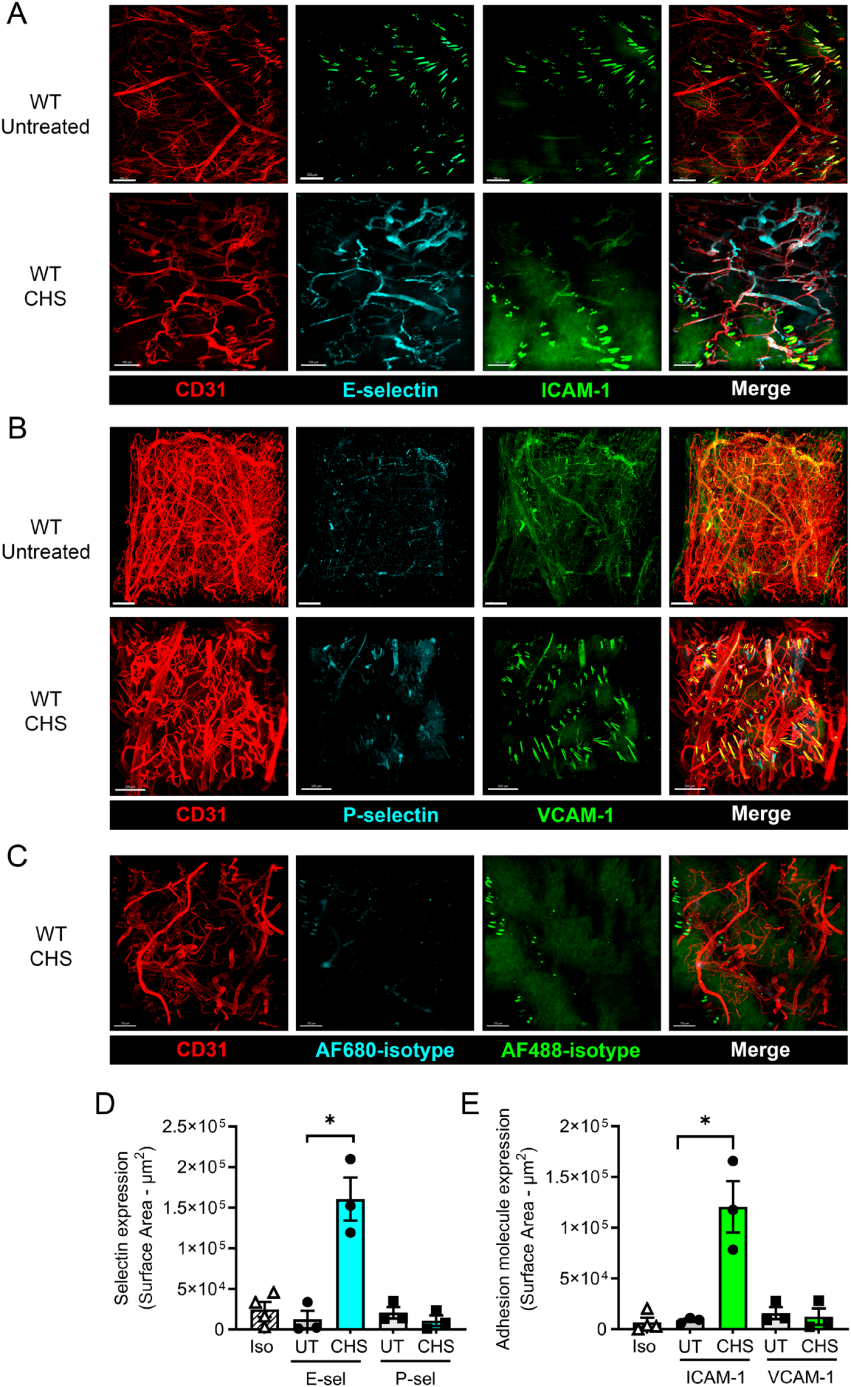
CHS induces upregulation of E‐selectin and ICAM‐1 in the dermal microvasculature. Adhesion molecule expression in the dermal microvasculature in a two‐challenge model of CHS was assessed in wild‐type (WT) C57BL/6 mice 4 h after the second challenge, via in vivo labelling with fluorochrome‐conjugated antibodies. Tissues underwent intravascular staining followed by exsanguination, fixation, clearing and imaging via multiphoton microscopy, with samples collected from CHS skin and untreated skin from the same mice. (A, B) Representative images of staining for CD31 (vasculature), E‐selectin and ICAM‐1 (A) or CD31, P‐selectin and VCAM‐1 (B) either in untreated skin (top row in A, B) or during CHS (bottom row in A, B). Images are maximum intensity projections through the skin, taken from the epithelial surface to ~400 μm depth in inflamed samples. Right‐hand panels show merged images. Scale bars = either 200 μm (A: WT untreated; B: WT CHS) or 150 μm (A: WT CHS; (B) WT Untreated). (C) Isotype control antibody staining in the dermal microvasculature during two challenge CHS. Mice underwent the two‐challenge model of CHS and received AF594‐anti‐CD31, along with AF680‐mouse IgG1κ (isotype control for RME‐1/anti‐E‐selectin) and AF488‐rat IgG2b (isotype control for YN1.1.7.4/anti‐ICAM‐1), and the skin was cleared and imaged as for the other samples. Images are shown for staining for CD31 (red), and the AF680‐conjugated (cyan) and AF488‐conjugated (green) isotype control antibodies. Scale bar = 150 μm. (D, E) Quantitation of adhesion molecule expression in untreated (UT) or CHS skin for E‐selectin and P‐selectin (D) or ICAM‐1 and VCAM‐1 (E). Data from samples treated with isotype control antibodies conjugated with AF680 (D) or AF488 (E) are also shown. Data are shown as mean ± sem with data for individual mice also shown. *N* = 3/group for adhesion molecules and 4/group for isotype controls. *p < 0.05 for comparison of UT and CHS (in the same animal) via paired *t*‐test for each adhesion molecule.

Our previous observations that interfering with Treg/endothelial cell interactions leads to increased adhesion of pro‐inflammatory leukocytes in two‐challenge CHS provided evidence that Tregs act to suppress endothelial cell adhesion molecule expression in this setting [33, 34]. To investigate this possibility, we established a novel imaging‐based technique for investigation of adhesion molecule expression in the dermal microvasculature. These experiments focused on E‐selectin, P‐selectin, ICAM‐1, and VCAM‐1, the endothelial adhesion molecules most likely to be involved in dermal leukocyte recruitment during CHS [22, 33, 43, 44]. Using anti‐adhesion molecule antibodies conjugated to non‐overlapping fluorochromes in combination with tissue clearing, we observed that two‐challenge CHS resulted in a significant increase in the surface area of endothelium positive for E‐selectin and ICAM‐1, while expression of P‐selectin and VCAM‐1 was unaltered (Figure 2A,B,D,E). Examination of uninflamed regions of skin in the same mice revealed that adhesion molecule staining was not greater than the level of staining seen in mice receiving isotype control antibodies (Figure 2C–E), indicating that constitutive adhesion molecule expression in the dermal microvasculature was below the sensitivity of this technique.

### 3.3 Tregs Control Endothelial Cell Adhesion Molecule Expression During CHS and in Untreated Skin

**FIGURE 3:**
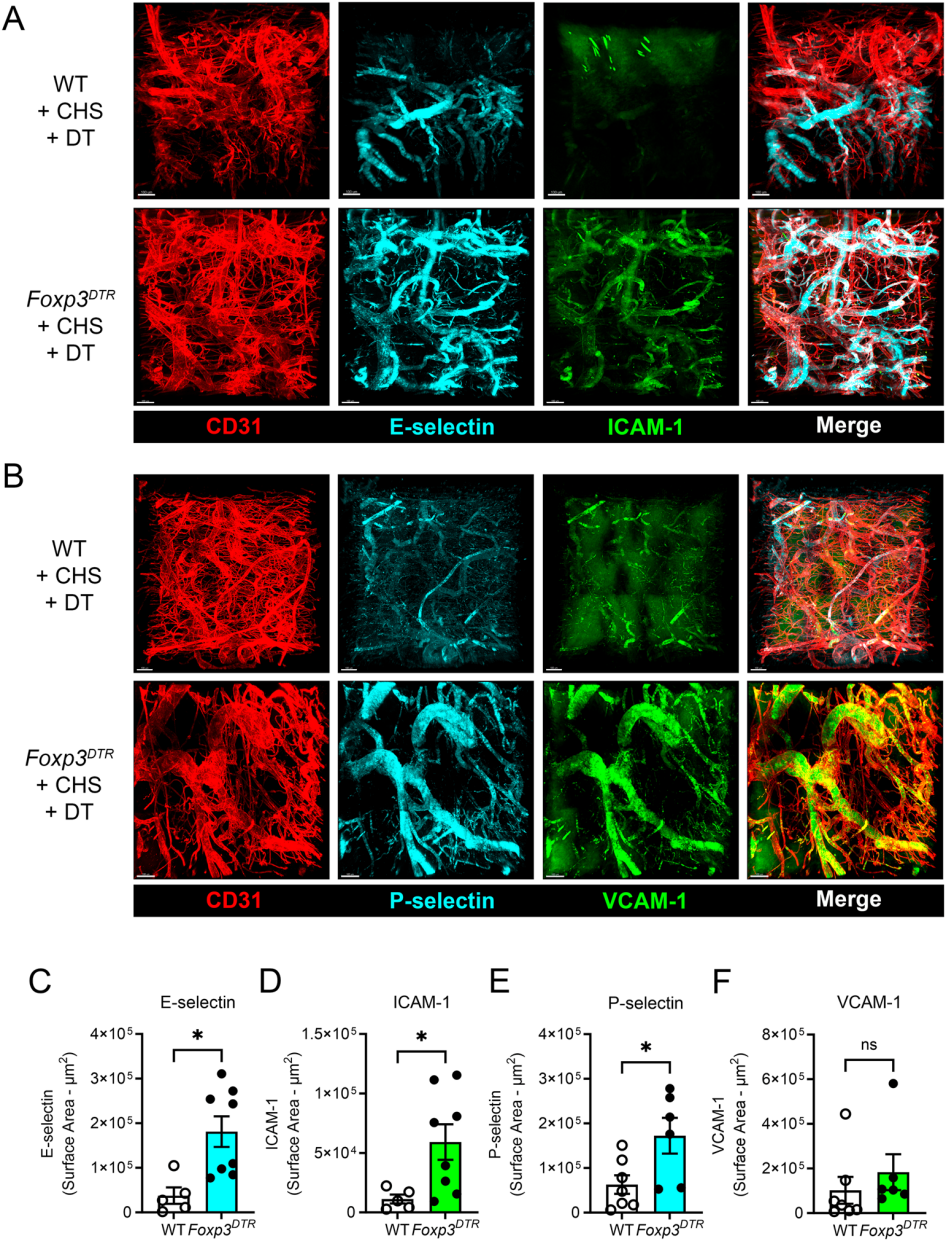
Treg depletion promotes increased expression of adhesion molecules in the dermal microvasculature during CHS. Adhesion molecule expression in the dermal microvasculature during two‐challenge CHS was assessed in wild‐type (WT) mice and *Foxp3* ^DTR^ mice treated with diphtheria toxin (DT) to deplete Tregs, commencing 24 h after the initial oxazolone challenge. Adhesion molecule expression was assessed 4 h after the second challenge as described in Figure 2. (A, B) Representative images of staining for CD31 (vasculature), E‐selectin and ICAM‐1 (A) or CD31, P‐selectin and VCAM‐1 (B) in DT‐treated wild‐type mice (WT + CHS + DT) or DT‐treated *Foxp3* ^DTR^ mice (*Foxp3* ^DTR^ + CHS + DT). Right‐hand panels show merged images. Scale bars = 100 μm. (C–F) Quantitation of adhesion molecule expression in DT‐treated wild‐type and *Foxp3* ^DTR^ mice undergoing CHS. Data are shown for E‐selectin (C), ICAM‐1 (D) P‐selectin (E) and VCAM‐1 (F). Data are shown as mean ± sem with data for individual mice also shown. *N* = 5 for WT (E‐selectin/ICAM‐1), 8 for *Foxp3* ^DTR^ (E‐selectin/ICAM‐1), 7 for WT (P‐selectin/VCAM‐1) and 6 for *Foxp3* ^DTR^ (P‐selectin/VCAM‐1). *p < 0.05 for comparisons shown via unpaired *t*‐test.

**FIGURE 4:**
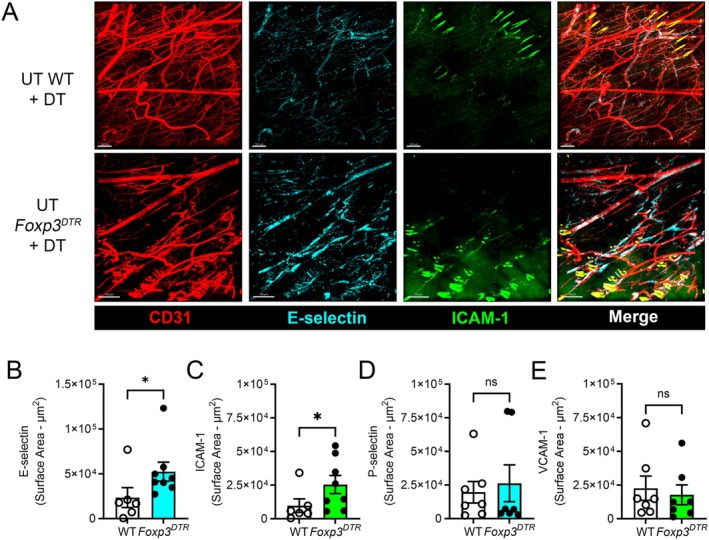
Treg depletion promotes increased expression of E‐selectin and ICAM‐1 in the uninflamed dermal microvasculature. Adhesion molecule expression in the dermal microvasculature of untreated skin was compared in Treg‐intact wild‐type mice and Treg‐depleted *Foxp3* ^DTR^ mice 28 h after initiation of Treg depletion. Adhesion molecule expression was assessed as described in Figure 2. (A) Representative images of staining for CD31 (vasculature), E‐selectin, and ICAM‐1 in untreated skin of DT‐treated wild‐type mice (UT WT + DT) or DT‐treated *Foxp3* ^DTR^ mice (UT *Foxp3* ^DTR^ + DT). Right‐hand panels show merged images. Scale bars = 100 μm (UT WT + DT) or 150 μm (UT *Foxp3* ^DTR^ + DT). (B–E) Quantitation of adhesion molecule expression in untreated skin of DT‐treated wild‐type (WT) and *Foxp3* ^DTR^ mice. Data are shown for E‐selectin (B), ICAM‐1 (C) P‐selectin (D) and VCAM‐1 (E). Data are shown as mean ± sem with data for individual mice also shown. *N* = 6 for WT (E‐selectin/ICAM‐1), 8 for *Foxp3* ^DTR^ (E‐selectin/ICAM‐1), 7 for WT (P‐selectin/VCAM‐1) and 7 for *Foxp3* ^DTR^ (P‐selectin/VCAM‐1). *p < 0.05 for comparisons shown via unpaired *t*‐test. ns, not significant.

To address the role of Tregs in controlling endothelial adhesion molecule expression, we next examined the two‐challenge CHS response in mice in which Tregs had been depleted by DT treatment during the challenge phase. Expression of E‐selectin, P‐selectin and ICAM‐1, but not VCAM‐1, were all significantly greater in skin of Treg‐depleted *Foxp3* ^DTR^ mice, when compared to DT‐treated wild‐type mice undergoing the CHS response (Figure 3A–F). Examination of regions of uninflamed skin from DT‐treated wild‐type and *Foxp3* ^DTR^ mice also revealed significant upregulation of E‐selectin and ICAM‐1, but not P‐selectin and VCAM‐1, in Treg‐depleted mice in otherwise uninflamed skin (Figure 4A–E). Together these findings demonstrate that Tregs control endothelial adhesion molecule expression both during two‐challenge CHS and under steady‐state conditions.

### 3.4 Extravascular Tregs Are Intimately Associated With the Dermal Vasculature Following a Second Hapten Challenge

**FIGURE 5:**
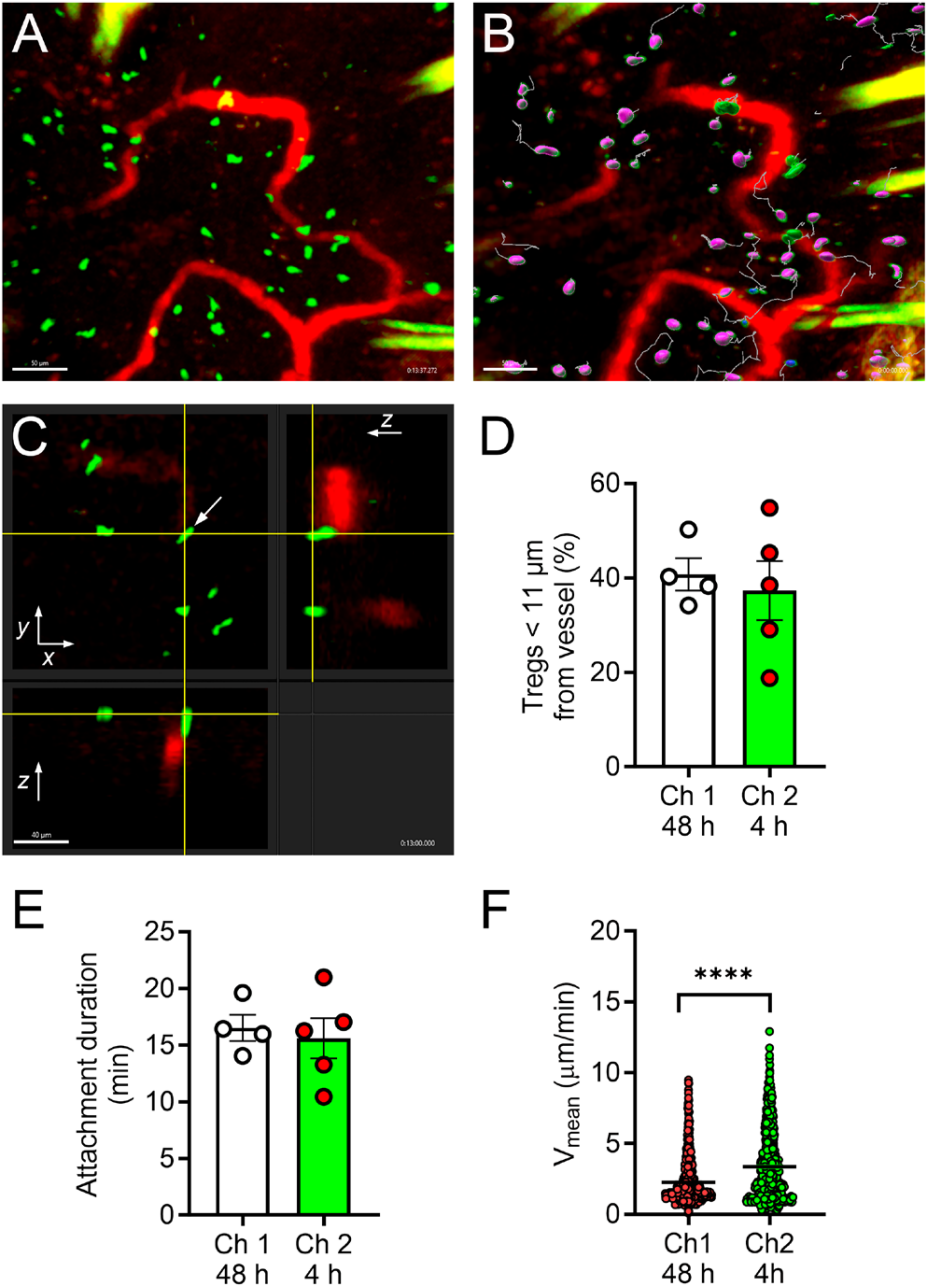
MP‐IVM reveals perivascular localisation of Tregs in two challenge CHS. Foxp3‐GFP Treg reporter mice underwent MP‐IVM during CHS, either 48 h after a single challenge (Ch 1, 48 h) or 4 h after a second challenge (Ch 2, 4 h). The position of Tregs relative to the dermal microvasculature was determined by labelling the microvasculature with AF680‐albumin. (A) Example MP‐IVM image of the upper dermis of a Foxp3‐GFP mouse, showing Tregs (green) adjacent to the microvasculature (red). (B) Image corresponding to (A) in which colored surfaces and migration tracks have been added to the Tregs. Tregs within 10 μm of a vessel are denoted by a green surface, while Tregs > 10 μm (~ one cell width) away from the microvasculature are denoted by a purple surface (still images from corresponding Videos S1 and S2). Scale bar = 50 μm. (C) Orthogonal view of a magnified region of Video S1 showing single *z*, *x* and *y* planes of the same field of view, containing a Treg (arrow) adjacent to a vessel. Yellow lines denote position of planes in each dimension, intersecting at the Treg. Scale bar = 40 μm. (D, E) Analysis of Treg localisation relative to the microvasculature at Ch 1, 48 h and Ch 2, 4 h, as determined using 30‐min MP‐IVM imaging periods. Data are shown for the % of Tregs that come to < 11 μm of the microvasculature (D), and the mean duration of this perivascular localisation (E). Data are shown as mean ± sem, with data for individual mice also shown. *N* = 4 mice/group. (F) Treg migration velocity at Ch 1, 48 h and Ch 2, 4 h, showing data for individual Tregs (Ch 1, 48 h—*n* = 590 Tregs from 4 mice; Ch 2, 4 h—*n* = 637 Tregs from 4 mice). Data are shown as mean ± sem. ****p < 0.0001 for comparison shown, via unpaired *t*‐test.

Our previous observations of Tregs in CHS revealed that while interfering with the intravascular interactions of Tregs enhanced inflammation, these interactions remained relatively infrequent, with adherent Tregs observed in only a low proportion of vessels at any single time [33, 34, 35]. In contrast, extravascular Tregs were highly abundant in the dermis, particularly at the time of the second challenge, raising the possibility that the extravascular Tregs and possibly recently transmigrated Tregs are responsible for control of endothelial function in this setting [41, 42]. In line with this, we reasoned that for extravascular Tregs to modulate endothelial function, they would need to be localized adjacent to the microvasculature. To explore this, we performed MP‐IVM of the skin of Foxp3‐GFP (Treg reporter) mice undergoing CHS, and visualized the microvasculature via labelling with AF680‐albumin (Figure 5). Both 48 h after a single challenge and 4 h after a second challenge, numerous Tregs were found adjacent to the microvasculature (Figure 5A–C and Videos S1 and S2). Indeed, approximately 40% of Tregs observed during the 30‐min imaging period came within 11 μm of the microvascular lumen (Figure 5D and Video S2). In addition, these vessel‐adjacent Tregs remained close to the vasculature for an average of 15 min, in some instances migrating along beside the microvessel (Figure 5E). Further analysis of Treg migration revealed that after the first challenge, Tregs were highly migratory, showing mean migration velocities of ~2.3 μm/min (Figure 5F), similar to our previous observations in CHS‐inflamed mice [41]. Four hours after a second challenge, Treg migration velocity was further increased to ~3.4 μm/min (Figure 5F). These observations reveal that Tregs undergo prolonged intimate associations with the microvasculature during CHS, while maintaining the highly migratory phenotype characteristic of CHS.

### 3.5 Regulation of Skin Inflammatory Gene Expression by Tregs During CHS

**FIGURE 6:**
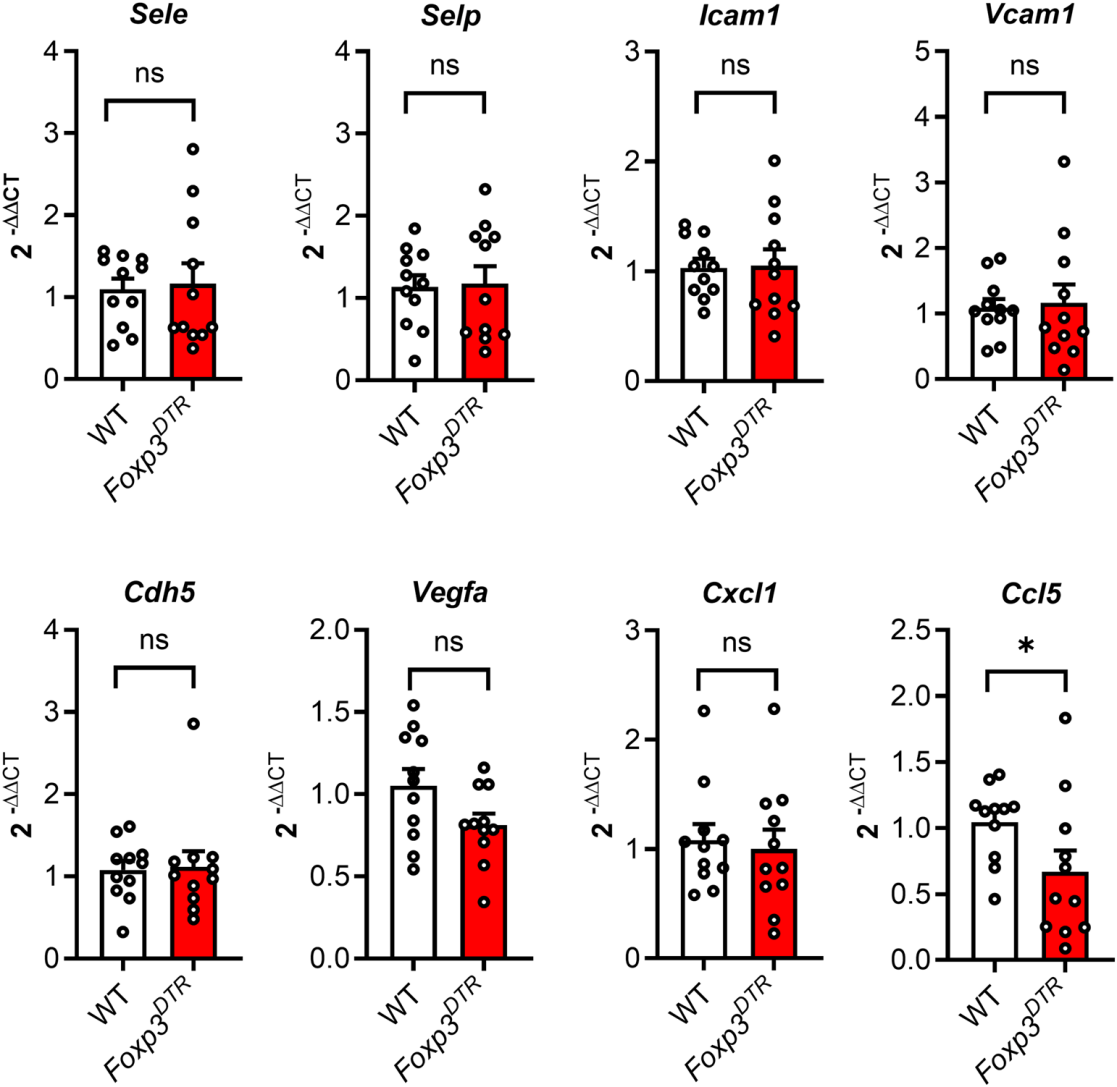
Effect of Treg depletion on inflammatory gene expression in skin during two‐challenge CHS. Gene expression in flank skin of mice undergoing two‐challenge CHS was assessed via qRT‐PCR, comparing Treg‐intact wild‐type (WT) mice and Treg‐depleted *Foxp3* ^DTR^ mice. Both strains of mice were treated with DT commencing 24 h after the initial oxazolone challenge, with Treg depletion confirmed in *Foxp3* ^DTR^ mice via flow cytometry, and samples collected 4 h after the second challenge. Data are shown for *Sele*, *Selp*, *Icam1*, *Vcam1*, *Cdh5*, *Vegfa*, *Cxcl1* and *Ccl5* (further data are shown in Table 1). Data (2^−ΔΔCT^) are shown as mean ± sem, with data for individual mice also shown. *N* = 11 mice/group. *p < 0.05 for comparisons shown via unpaired *t*‐test. NS, not significant.

Alterations in microvascular adhesion molecule expression stemming from removal of Tregs may stem from changes in gene transcription or alterations in the retention of adhesion molecule protein on the endothelial cell surface. To investigate this, we asked whether gene transcription for endothelial adhesion molecules was altered by removal of Tregs. This was achieved using qRT‐PCR to assess expression of the adhesion molecules examined above, as well as a panel of inflammatory genes in the inflamed skin, comparing wild‐type and Treg‐depleted *Foxp3* ^DTR^ mice during the CHS response (Figure 6 and Table 1). In contrast to the changes in protein expression on the endothelial surface observed with Treg depletion (Figure 3), no differences in mRNA expression of the genes for adhesion molecules E‐selectin (*Sele*), P‐selectin (*Selp*), ICAM‐1 (*Icam1*) and VCAM‐1 (*Vcam1*) were detected. Similar observations were made for a range of chemokines, cytokines, and inflammatory molecules with potential roles in the microvasculature, except for *Ccl5*, which was reduced in Treg‐depleted mice (Figure 6 and Table 1). These findings indicate that in this setting, the effect of Tregs on adhesion molecule expression did not occur at the level of gene transcription, and therefore is likely to have stemmed from altered retention of protein on the endothelial cell surface.

**TABLE 1:** Effect of Treg depletion on inflammatory gene expression in skin during two‐challenge CHS.^a^

Gene	WT	*Foxp3* ^DTR^
*Ccl2*	1.52 ± 0.39	1.23 ± 0.31
*Cxcl9*	1.13 ± 0.18	0.88 ± 0.23
*Cxcl10*	1.25 ± 0.26	1.33 ± 0.31
*Cx3cl1*	1.14 ± 0.18	1.31 ± 0.19
*H2‐Ab1*	1.08 ± 0.12	1.06 ± 0.20
*Tnf*	1.11 ± 0.14	0.85 ± 0.12
*Ifng*	1.52 ± 0.39	1.23 ± 0.31
*Il4*	1.30 ± 0.28	1.78 ± 0.39
*Csf2*	1.14 ± 0.17	0.90 ± 0.16
*Cd274*	1.07 ± 0.12	0.80 ± 0.13
*Ido*	1.50 ± 0.45	1.43 ± 0.60
*Nos2*	1.29 ± 0.27	0.66 ± 0.15
*Nos3*	1.05 ± 0.1	1.11 ± 0.13
*Smad2*	1.10 ± 0.16	1.17 ± 0.07

*Note:* Comparing Treg‐intact wild‐type (WT) mice and Treg‐depleted *Foxp3* ^DTR^ mice as per Figure 6. Data (2^−ΔΔCT^) are shown as mean ± sem Both strains of mice were treated with DT commencing 24 h after the initial oxazolone challenge, with Treg depletion confirmed in *Foxp3* ^DTR^ mice via flow cytometry, and samples collected 4 h after the second challenge. *N* = 11 mice/group.

^a^ Gene expression in flank skin of mice undergoing two‐challenge CHS was assessed via qRT‐PCR.

## 4 Discussion

While the effects of Tregs on other immune cell populations such as effector T cells and dendritic cells have been investigated in detail, less is known about the actions of Tregs on the microvascular endothelium. Recent analyses of a model of lung cancer demonstrated that endothelial cells respond to elimination of Tregs within 48 h, showing alterations in gene expression well before T cells, the conventional targets of Treg activity [30]. These observations provide clear evidence that endothelial cells are an important early target of the actions of Tregs. We have previously observed that interfering with Treg‐endothelial interactions promotes inflammation in a two‐challenge CHS model in which resolution of the inflammatory response is accelerated [31, 32, 33, 34, 35]. The absence of Tregs during CHS has also been associated with reduced control of the endothelial barrier and increased leukocyte recruitment to the skin [15, 16, 17, 18]. Here we show that in a two‐challenge CHS model, intradermal Tregs surround the dermal microvasculature and undergo prolonged dynamic associations with it. Treg depletion resulted in increased expression of E‐selectin, P‐selectin and ICAM‐1 on the endothelial surface, along with increased tissue swelling consistent with loss of endothelial barrier function. This effect occurred in the absence of an alteration in transcription of the genes for these adhesion molecules, indicating that this effect occurred at the level of protein expression on the endothelial cell surface. Together these data demonstrate that the dermal microvascular endothelium is an important target of the anti‐inflammatory actions of Tregs.

Tregs possess a wide variety of mechanisms for regulating pro‐inflammatory actions of target cells, some dependent on cell–cell contact and others being mediated by generation of soluble mediators [19]. Furthermore, their capacity to regulate skin inflammation has been linked to their ability to undergo recruitment and retention in this organ [10, 11, 12, 13]. Therefore, we reasoned that an effect of Tregs on dermal microvascular endothelial cells would be reliant on colocalization of these two cell types. Our previous analyses of the actions of intravascular Tregs during the CHS response revealed that some Tregs undergo prolonged intravascular attachment to the endothelium in dermal post‐capillary venules, demonstrating one form of colocalization of these cell types [35]. Here we extended this by examining the localisation of extravascular Tregs. At both 48 h after a single challenge, and 4 h after a second challenge, a significant proportion of the extravascular Tregs were located adjacent to the dermal microvasculature. Moreover, despite their highly migratory nature [41, 42], intradermal Tregs remained in this perivascular location for at least 15 min. In our previous studies of the two‐challenge CHS model, inhibiting Treg interactions with the endothelium resulted in evidence of endothelial activation. From these observations, it is reasonable to hypothesize that the associations of Tregs with the microvasculature observed here and previously underpin their ability to modulate endothelial cell function.

Induced Tregs (iTregs) are capable of suppressing endothelial cell adhesion molecule expression in vitro via induction of contact‐independent TGFβ‐dependent signaling [27]. Tregs from human cancer patients can also suppress lymphocyte adhesion and transendothelial migration [28]. However, notably in the latter case, the mechanism is contact‐dependent and TGFβ/IL‐10‐independent. In vivo studies in CHS have shown that adoptively‐transferred Tregs can reduce inflammation‐associated leukocyte adhesion in skin microvessels and strengthen endothelial barrier function via mechanisms including secretion of IL‐10 and CD39‐dependent adenosine generation [15, 16, 18]. Together, these studies demonstrate that mechanisms of Treg‐driven regulation of endothelial function differ in different circumstances. Here, as opposed to transferring Tregs, we eliminated endogenous Tregs during the CHS effector phase, in this way, demonstrating the actions of Tregs normally recruited to the skin during the CHS response. Further investigation will be required to determine whether the same Treg‐derived soluble mediators or contact‐dependent mechanisms are responsible for the Treg‐dependent effects observed.

Endothelial cells play a central role in the skin inflammatory response by promoting recruitment of inflammatory leukocytes via expression of P‐selectin, E‐selectin and ICAM‐1 [21, 22, 23]. Therefore, we used adhesion molecule expression on the endothelial surface as a readout of endothelial cell activation and the effect of elimination of Tregs, showing that Tregs act to restrict upregulation of P‐selectin, E‐selectin and ICAM‐1 during CHS. Notably, the augmented adhesion molecule expression following Treg depletion occurred in the absence of changes in mRNA encoding the respective genes. This indicates that Tregs suppress expression of these molecules on the endothelial cell surface via post‐translational mechanisms. Both P‐selectin and E‐selectin are down‐regulated by internalization, while all of the adhesion molecules studied here are also released in soluble form by endothelial cells [45, 46, 47]. The present observations provide evidence that Tregs restrict adhesion molecule expression by impacting one or more of these mechanisms of downregulation of adhesion molecule protein.

Dermal postcapillary venules have been hypothesized to constitutively express E‐selectin, facilitating skin homing of lymphocytes expressing the cutaneous lymphocyte antigen (CLA) [48, 49]. This is supported by observations from steady‐state human and porcine skin showing a low constitutive level of E‐selectin expression [50, 51]. Given these observations, the minimal level of E‐selectin detected in uninflamed mouse skin in the present study was arguably unexpected. However, previous studies of mouse skin in which E‐selectin expression was assessed using intravenously‐administered antibodies also showed minimal constitutive E‐selectin expression [44, 52]. Furthermore, intravital microscopy studies of mouse skin have demonstrated that the bulk of the leukocyte rolling in uninflamed dermal venules can be eliminated via inhibition of P‐selectin, indicating that the functional contribution of E‐selectin in resting mouse skin is minimal [34, 53]. Nevertheless, the results of both the present study and previous work clearly demonstrate that upon inflammatory stimulation, the dermal microvasculature preferentially upregulates E‐selectin as compared to P‐selectin, a response distinct from that seen in other organs [53]. The mechanism underpinning this particular propensity of the inflamed dermal microvasculature is unclear, although the present finding that, upon removal of Tregs, P‐selectin is strongly upregulated in skin blood vessels suggests that dermal Tregs may contribute.

In conclusion, these studies indicate that an important pathway whereby Tregs control inflammatory outcomes in the skin during two‐challenge CHS is via suppression of endothelial adhesion molecule expression. The observation that Tregs are enriched immediately adjacent to the microvasculature during this phase of CHS supports the concept that this is an important mechanism whereby skin inflammation is regulated by Tregs. While these findings raise the possibility that Tregs mediate this effect by directly affecting endothelial function, Tregs can also affect the function of other cells in the inflammatory milieu, such as macrophages and fibroblasts [30]. Given this, it is conceivable that the effect of Tregs on endothelial cells is indirect, and occurs via impacts on an intermediary cell type. Further investigation will be required to elucidate the molecular mechanisms whereby Tregs achieve this effect.

### 4.1 Perspectives

This work shows that Tregs in inflamed skin are enriched around the vasculature and work to suppress adhesion molecule expression. Notably, Tregs also mediate this effect in otherwise uninflamed skin. Improving our understanding of how Tregs control skin inflammation has the potential to inform the development of novel approaches to control diseases including allergic contact dermatitis and psoriasis.

## Conflicts of Interest

The authors declare no conflicts of interest.

## Supporting information


**Table S1:** Genes and assay numbers used in Taqman qRT‐PCR assays.

**Video S1:** Multiphoton intravital microscopy (MP‐IVM) of challenged skin of a Foxp3‐GFP Treg reporter mouse undergoing CHS 4 h after a second challenge. Tregs (green) are visible adjacent to the dermal microvasculature (labeled with AF680‐albumin—red). The video rotates to enable the position of the Tregs relative to the microvasculature to be discerned more readily (see also Figure 5). Yellow structures are hairs. Video duration = 29 min in real time.

**Video S2:** The same video sequence as for Video S1 in which colored surfaces and migration tracks have been added to the Tregs. Tregs within 10 μm of a vessel are denoted by a green surface, while Tregs > 10 μm away from the microvasculature are denoted by a purple surface. Yellow structures are hairs. Video duration = 29 min in real time.

## Data Availability

No datasets were generated or analyzed during this study.
