# Supplementary material for: Regulatory T Cells Control Vascular Adhesion Molecule Expression in Skin Under Inflammatory and Homeostatic Conditions
Source: Microcirculation. 2025 Jun 29;32(5):e70017. doi: 10.1111/micc.70017 (PMC12206477; doi:10.1111/micc.70017)
Supplement: Supplementary file 1 — Table S1: Genes and assay numbers used in Taqman qRT‐PCR assays. [file MICC-32-e70017-s002.docx]

**Supplementary Table S1: Genes and assay numbers used in Taqman qRT-PCR assays**

| **Gene name** | **Taqman assay number** |
| --- | --- |
| *Actb* | Mm00607939_s1 |
| *Sele* | Mm00441278_m1 |
| *Selp* | Mm00441295_m1 |
| *Icam1* | Mm00516023_m1 |
| *Vcam1* | Mm01320970_m1 |
| *Cdh5* | Mm00486938_m1 |
| *H2-Ab1* | Mm00439216_m1 |
| *Vegfa* | Mm00437306_m1 |
| *Ifng* | Mm01168134_m1 |
| *Tnf* | Mm00443258_m1 |
| *Il4* | Mm00445259_m1 |
| *Csf2* | Mm00438328_m1 |
| *KC/Cxcl1* | Mm04207460_m1 |
| *Cxcl9* | Mm00434946_m1 |
| *Cxcl10* | Mm00445235_m1 |
| *Ccl2* | Mm00441242_m1 |
| *Ccl5* | Mm01302427_m1 |
| *Cx3cl1* | Mm00436454_m1 |
| *Cd274* | Mm03048248_m1 |
| *Nos2* | Mm00440502_m1 |
| *Nos3* | Mm00435217_m1 |
| *Smpd3* | Mm00491359_m1 |
| *Smad2* | Mm00487530_m1 |
| *Ido1* | Mm00492586_m1 |
